# Promoting healthy eating in Latin American restaurants: a qualitative survey of views held by owners and staff

**DOI:** 10.1186/s12889-022-13294-7

**Published:** 2022-04-27

**Authors:** Melissa Fuster, Rosa Abreu-Runkle, Margaret A. Handley, Donald Rose, Michelle A. Rodriguez, Emily G. Dimond, Brian Elbel, Terry T. K. Huang

**Affiliations:** 1 Department of Social, Behavioral, and Population Sciences, Tulane University School of Public Health and Tropical Medicine, New Orleans, LA USA; 2 Department of Hospitality Management, School of Professional Studies, New York City College of Technology, City University of New York, New York, NY USA; 3 Department of Epidemiology and Biostatistics, School of Medicine, University of California, San Francisco, CA USA; 4 City University of New York Graduate School of Public Health and Health Policy, New York, NY USA; 5 Department of Population Health, New York University Grossman School of Medicine, New York, NY USA; 6 Wagner Graduate School of Public Service, New York University, New York, NY USA; 7 Department of Community Health and Social Sciences and Center for Systems and Community Design, City University of New York Graduate School of Public Health and Health Policy, New York, NY USA

**Keywords:** Food environment, Restaurants, Latin/Hispanic, Nutrition, Food access, Health equity, Qualitative research

## Abstract

**Background:**

Restaurants, particularly independently-owned ones that serve immigrant communities, are important community institutions in the promotion of dietary health. Yet, these restaurants remain under-researched, preventing meaningful collaborations with the public health sector for healthier community food environments. This research aimed to examine levels of acceptability of healthy eating promotion strategies (HEPS) in independently-owned Latin American restaurants (LARs) and identify resource needs for implementing HEPS in LARs.

**Methods:**

We completed semi-structured, online discussions with LAR owners and staff (*n* = 20), predominantly from New York City (NYC), to examine current engagement, acceptability, potential barriers, and resource needs for the implementation of HEPS. Verbatim transcripts were analyzed independently by two coders using Dedoose, applying sentiment weighting to denote levels of acceptability for identified HEPS (1 = low, 2 = medium/neutral, 3 = high). Content analysis was used to examine factors associated with HEPS levels of acceptability and resource needs, including the influence of the Coronavirus pandemic (COVID-19).

**Results:**

The most acceptable HEPS was menu highlights of healthier items (mean rating = 2.8), followed by promotion of healthier items (mean rating = 2.7), increasing healthy offerings (mean rating = 2.6), nutrition information on the menu (mean rating = 2.3), and reduced portions (mean rating = 1.7). Acceptability was associated with factors related to perceived demand, revenue, and logistical constraints. COVID-19 had a mixed influence on HEPS engagement and acceptability. Identified resource needs to engage in HEPS included nutrition knowledge, additional expertise (e.g., design, social media, culinary skills), and assistance with food suppliers and other restaurant operational logistics. Respondents also identified potential policy incentives.

**Conclusions:**

LARs can positively influence eating behaviors but doing so requires balancing public health goals and business profitability. LARs also faced various constraints that require different levels of assistance and resources, underscoring the need for innovative engagement approaches, including incentives, to promote these changes.

## Background

Hispanics have a higher burden of diet-related health conditions and risk factors [1]. Among Hispanics, as in the case of the population at large, the consumption of foods away from home is prevalent and has been associated with decreased diet quality and cardiovascular disease risk factors [2–4]. This indicates a need to engage the sector in promoting healthy eating to combat prevalent diet-related conditions. Restaurants are starting to implement changes that can potentially facilitate healthier eating, including adding nutrition information to menus, increasing the availability of healthier options, and promoting them. However, these efforts mostly occur in corporate restaurants; less activity has been observed in independently-owned restaurants, especially those serving ethnic cuisines [5], such as Latin American restaurants (LARs). This omission is pertinent because LARs comprise an increasingly important sector within the larger restaurant landscape in the United States (US). The Hispanic population in the US is projected to increase by 61% in the next 30 years, from 60.5 million in 2019 to 99.8 million in 2050 [6, 7]. The growing Hispanic population comes with an increased presence in businesses serving the community and beyond, including LARs, given the sector’s significance as an entryway to the workforce and opportunities for entrepreneurship [8]. There are over 120,000 LARs in the US, most of which are independently owned, and Mexican restaurants alone make up 8% of all US restaurants [9, 10]. Beyond serving the Hispanic community, LARs are increasingly popular among the public. According to the National Restaurant Association, 80% of consumers eat at a restaurant serving ethnic cuisine at least once a month [11], making collaborations with the sector potentially impactful beyond Hispanic populations.

Emerging research in small, non-corporate restaurants demonstrates interventions have the potential to increase the consumption of healthier options, yet the evidence is still limited due to a paucity of research in this sector [12]. Research is even more limited regarding eateries serving immigrant communities [12, 13]. These establishments are essential, as they have the potential to serve a role beyond the provision of food. Ethnic restaurants are venues for social interactions and economic opportunity [8, 14–16]. However, they face unique challenges related to cultural and language differences, as well as staff and owners’ immigration status, which may prevent these restaurants from accessing economic and other resources for their businesses. To our knowledge, few intervention studies have engaged LARs [17], and more information is needed to understand this sub-sector’s perspective concerning potential healthy eating promotion strategies and the barriers that may uniquely affect LARs.

The present study addressed this research gap by examining the acceptability and potential barriers for the implementation of healthy eating promotion strategies (HEPS) among non-corporate LAR owners and staff and identifying resource needs for the implementation of selected HEPS.

## Methods

### Study context

Data were collected between October 2020 and June 2021 through virtual interviews with LAR owners and staff. The virtual setting for our study was the result of adaptations in response to the COVID-19 pandemic, a critical contextual consideration for our study. The onset of the pandemic included restrictions to indoor dining and staff shortages, where the US restaurant industry suffered tremendous losses, and the number of employees dropped by 17% from 2019 to 2020, to the lowest number seen in the past decade [18]. The data collection period encompassed different stages in the pandemic, including the gradual lifting of restrictions and the gradual initial recovery due to vaccination efforts [19]. Across the US, restaurants were facing an uncertain period full of ongoing adaptations, including changes to operations and staffing, with potential implications for their acceptance of HEPS – aspects that were acknowledged during our data collection period.

### Sampling and recruitment

This research used a non-random purposive sample, targeting adults (18 years of age or over) with experience working in LARs. Our inclusion criteria included being a self-identified LAR owner, manager, waitstaff, and/or cook/chef and working currently or within the past 6 months in a restaurant that served Latin American cuisine (e.g., Mexican, Puerto Rican, etc.). Outreach was conducted using the researchers’ existing community networks and social media. We created a study website and social media accounts (i.e., Facebook, Twitter, and Instagram) to disseminate study recruitment announcements, using hashtags with bilingual postings relevant to the target population (e.g., #LatinRestaurant, #Restaurant, and cuisine-specific hashtags, such as #PuertoRicanRestaurant). Interested participants completed a screening survey to determine eligibility, facilitate scheduling, and collect initial information about the participants and the restaurants. The survey was available in English and Spanish. Eligible participants were scheduled for an interview via e-mail. They were provided with the study information, informed consent form, and instructions to access the virtual meeting via Zoom. Upon completing the discussion, participants received a $50 e-gift card. As an added incentive for owners, their restaurant was included in the project’s social media promotion efforts.

### Data collection

The screening survey collected initial demographic information and restaurant characteristics. The demographic information included participant race/ethnicity and professional involvement in the restaurant industry, such as their role(s) and years of experience. Restaurant characteristics included location, main cuisine(s) served, price range, COVID-19 impact on operations, and current HEPS engagement.

The discussions were originally planned as group interviews. However, we adapted to include individual interviews, given challenging issues with scheduling and difficulty recruiting participants. We carried out two group interviews of 3 participants, each lasting approximately 1.5 hours, and 14 individual interviews lasting on average 53 minutes. The group interview participants shared the same main role in a restaurant. One group included owners, and the second included chefs.

The interviews were led by a trained facilitator (MF, study PI), accompanied by at least one co-facilitator and note-taker. The interviews took place in English or Spanish, depending on participant preference. We developed a semi-structured interview guide informed by an extensive review of the literature, including relevant articles and reports on industry perspectives and practices concerning HEPS [5]. The guide included four sections: (1) initial questions, asking respondents to share their experience in the restaurant industry and, for owners, the restaurant concept and opening process; (2) experience with COVID-19, including past and potential future modifications to the business model; (3) experience with and perception of HEPS, including pre-defined HEPS (i.e. the provision of healthier options, menu highlights of healthier items, portion size reduction/options, and nutrition information on menus) and providing the opportunity for other possibilities raised by participants; and (4) resource needs to engage in HEPS. The guide was tailored to participants’ role in a restaurant (i.e., owner, chef/cook, or server) and experience with HEPS, as reported in the screening survey. After each interview, the research team debriefed to ascertain (1) the main findings from the discussion; (2) concepts or ideas that were repeated from past discussions; (3) new insights gained; and (4) areas or questions that remained unanswered or that emerged from the discussion. The debriefing notes were used to identify areas for follow-up in forthcoming interviews and to establish the data saturation point, where no new themes of ideas were identified regarding the main focus of the study [20].

All methods and procedures were carried out following relevant guidelines. The research was reviewed and deemed exempt by the City University of New York Institutional Review Board, as research involving interview procedures with minimal or no risk to participants.

### Data analysis

Audio files were transcribed verbatim, translated as needed, and analyzed using Dedoose (version 9.0.15). We used content analysis through a mix of deductive and inductive approaches [21], focused on factors associated with HEPS acceptability levels. HEPS acceptability levels were examined using sentiment analysis via code weighting. Sentiment weighting involves the application of a value scale to a code to account for intensity, adding depth to content analysis [22]. We used a three-point scale (1–3) to rate the level of acceptability for commonly implemented HEPS, where 1 was used for excerpts denoting low acceptability and 3 was used for high acceptability. A middle value of 2 was used for neutral or medium acceptability, where the respondents discussed the HEPS with an ambivalent or neutral tone. Open coding was used to develop an initial list of codes for the factors associated with level of acceptability and resource needs for each strategy. The initial code list was generated independently by two research assistants, and then refined by the study PI (MF) and a lead team member (MAR). The open codes were revised and consolidated via team meetings, leading to a code book that was entered in Dedoose. All transcripts were then independently coded by two trained research assistants. A lead team (MAR) member reviewed all coding, and discrepancies were resolved in weekly team meetings, including the study PI (MF).

We engaged in data validation procedures via member checks and data triangulation [21]. Member checks included incorporating an expert in restaurant management as part of the research team and sharing emerging results with participants for verification during the data collection period. Triangulation was used by examining findings against findings gathered through a scoping review of the literature, examining restaurant engagement in healthy eating promotion strategies, including barriers and facilitators [5].

## Results

### Sample description

A total of 27 respondents completed the screening survey, and all were invited to participate in the discussions. Of those, 20 participated in the study, representing 13 restaurants (Table 1). Most study participants self-identified as Hispanic (*n* = 17) and more than half of the participants (*n* = 13) had 10 years or more of experience on the industry. Close to half of the participants were restaurant owners (*n* = 9), with the rest closely split between chefs (*n* = 6) and servers (*n* = 5) (Table 1). Only three participants were not currently working at a restaurant at the time of the interview, having left their positions as chefs for reasons related to COVID-19. Among the 7 that signed up and did not participate, all identified as Hispanic, including three owners and five servers, and all were working in the industry at the time. While we followed up with these participants, we were unable to ascertain the individual reasons for the no-shows.

**Table 1:** Characteristics of study participants

**I. Respondent Characteristics**	**Frequency (*n*** **= 20)**	**Percentage (%)**
Race/Ethnicity		
Hispanic	17	85
Non-Hispanic White	3	15
Years in Restaurant Industry		
1–3 years	3	15
4–6 years	2	10
7–9 years	2	10
10 years or more	13	65
Main role in restaurant		
Owner	6	30
Chef-Owner	3	15
Chef	6	30
Server/Front-of-House	5	25
**II. Characteristics of Restaurants Represented**	**Frequency (*n*** **= 13)**	**Percentage (%)**
Location		
New York City	10	77
Puerto Rico	2	15
Florida	1	8
Cuisine served		
Mexican	7	54
Latin Caribbean	4	31
South American	2	15
Restaurant type		
Counter-Style/Fast Casual	3	23
Full Service	10	77
Price Range (per customer)		
$ (less than $10)	0	0
$$ ($11–30)	9	69
$$$ ($31–60)	3	23
$$$$ (more than $61)	1	8

Most of the restaurants were in New York City (NYC, *n* = 10) and were classified as full-service (*n* = 10). More than half of the restaurants served Mexican food (*n* = 7), whereas the rest served Latin Caribbean foods (one Cuban and three Puerto Rican) or South American (*n* = 2, Peruvian and Uruguayan). The price range per customer at these restaurants varied, with most restaurants (*n* = 9) in the $11–30 price range, as reported by participants (Table 1). Most of the restaurants had a single location, except for two (one with two locations and a second, family-owned restaurant, with 14 locations, as of June 2021).

### Healthy Eating Promotion Strategies (HEPS): acceptability, barriers, and facilitators

HEPS used in this research are summarized in Table 2. They are ordered by the mean acceptability sentiment rating and discussed here in that order. For each strategy, we present an overview of the strategy, followed by factors associated with levels of acceptability. The next section compiles the resource needs to engage in the strategies.

**Table 2:** HEPS acceptability, prevalence, and illustrative excerpts

Strategy	Mean rating	Respondents with Previous Experience with given HEPS ***n*** (%)	Illustrative Excerpt		
			High (Rating = 3)	Neutral (Rating = 2)	Low (Rating = 1)
Menu highlights of healthy choices (e.g., Healthy markers on menus, Healthy menu sections)	2.8	6 (30%)	“Veganism will be good to show [on the menu]. Maybe it will give us a broader [audience]. We’ll be able to market to other customers we don’t reach [because] they don’t relate Mexican food with healthier food.” – Waitstaff, fast-casual Mexican Restaurant, NYC	[None]	“You don’t want to say healthy options because where are the other options, are the other options unhealthy?” - Chef, full-service Mexican restaurant, NYC
Promotion of healthier choices (e.g., Social media promotion, Server suggestions)	2.7	4 (20%)	“You don’t really need to make a major change on the menu, but with the power that we have nowadays with the social media, […] showing the dish, a nice picture [and] include that this is vegan or let’s include that seafood is important on our menu.” – Waitstaff, fast-casual Mexican restaurant	“We’ve asked waitstaff to highlight, whatever we want to highlight at that point. Sometimes it could be chimichanga and sometimes it can be a vegan option.” -Owner, full-service Mexican restaurant, NYC	“Why go the extra mile […] doing all this creative work, training anyone to present something [healthier] that is just as Mexican but is not going to sell?”– Chef, full-service Mexican restaurant
Provision of healthy options (e.g., vegetarian alternatives, vegetable dishes)	2.6	20 (100%)	“I believe that many people […] are also adding healthier choices to their menus. With everything that is happening now, with everything that is happening in the world, people want to live more years. They want to be healthy.” – Owner, counter-style Peruvian restaurant, NYC	“I think that there’s certain things that there’s really not any way of making it healthier. For instance, the lamb is a very, very fatty piece of meat, as well as the pork belly. It is what it is, but there are other things that can be modified to make them healthier. Small things, like the salad, if you would rather not have cheese, or the enchiladas, you can take the dairy away.”- Chef, full-service Mexican restaurant, NYC	“I can’t have [a refrigerator] full of vegetables and nobody necessarily orders the salad. [...] it’s not a consistent product and it can be expensive [...] we’re a restaurant and, to a point, we need to make the money. That’s how we look at it.” Chef, counter-style Puerto Rican restaurant NYC
Providing nutrition information (e.g., Calorie labeling)	2.3	2 (10%)	“I think it would be very acceptable, I really do. Nowadays, everyone – myself included – is counting the calories or looking to eat less carbs, trying to stick to a balanced diet.” - Waitstaff, full-service Cuban restaurant, Miami, FL	“I agree with the calories but I mean the change of mind for the society here in the US especially, it’s a difficult task.” – Owner, full-service Uruguayan restaurant, NYC.	“I feel that the type of restaurant that we are, it would take some serious effort probably to hire someone to actually break down and analyze the recipes.” -Chef, full-service Mexican restaurant, NYC
Reduced portion size(e.g., smaller portions, offering half-portion options)	1.7	5 (25%)	“I think it’s really helpful, the half portioning of some dishes. That’s something great when you go to eat that you don’t have to eat the entire thing.” – Owner, full-service Mexican restaurant, NYC	[None]	“I don’t see clients looking at the menu for half of a *vaca frita* [fried steak] or half the rice, I don’t see it. I believe that a person who comes to a Cuban restaurant, in general, almost in all the Cuban restaurants, they know they are going there to eat and eat plenty.” – Waitstaff, full-service Cuban restaurant, Miami, FL

#### Menu highlights

Menu highlights encompassed the use of special markers on menus (e.g., “V” for vegetarian), the grouping of healthy options in particular sections, or the use of a separate menu for healthy options. This strategy was largely viewed as acceptable across respondents. Acceptance was driven by two main factors: customer convenience by simplifying food choices and marketing potential to satisfy a perceived demand for healthier options, with the potential to expand the customer base (Table 2). Low acceptability came from chefs. One argued that labeling or marking certain offerings as healthy may imply that the rest of the menu is not healthy, while another noted that healthy menu sections might add a feeling of exclusion for customers interested in healthier offerings, as being relegated to the corner of a menu. Healthy sections are regularly included at the end of menus, diminishing their importance within the rest of the offerings and potentially decreasing their effectiveness in promoting healthier choices, as customers may be enticed by the less healthy options presented early in a menu.

#### Promotion of healthier options

The promotion of existing healthier options (Table 2) was discussed mainly as promotion via social media, as the primary medium for marketing, with increased importance after the onset of COVID-19. In addition, we also inquired about the promotion of healthier items by servers recommending these items to customers. The relatively high acceptability of this strategy was associated with the potential to better market the restaurant and because it offered an alternative to menu highlights, not requiring menu redesigns or the need to develop online menus, thus reducing menu production costs. Low acceptability was found among chefs and servers, who expressed concerns about customer reaction. For example, customers might react poorly to staff or the restaurant if server recommendations of healthy items triggered negative emotions among customers or otherwise offended them. Additionally, as noted by one chef, promotion efforts tended to focus on top sellers, not seeing healthier items in this category (Table 2).

#### Providing healthier food options

All the respondents came from restaurants that offered some potentially healthful options (Table 2), including green salads, vegetarian alternatives, and seafood options. This strategy was discussed based on their experience with current offerings and the potential for expanding these options. COVID-19 forced the restaurants to adapt their menus, temporarily taking out both healthier (e.g., green salads) and less healthy offerings (e.g., fried snacks). The adaptations were in response to shifts to takeout and delivery, issues with the food supply, and reduced staff due to capacity restrictions, concerns for staff health, and the inability to keep the payroll. The shift to take out and delivery forced some restaurants to experiment with this service mode for the first time, pushing them to rethink menus and packaging to retain food quality. These experiences with menu changes served as a starting point to discuss potential increases in healthier offerings.

Acceptability for this strategy was associated with factors that could increase profit, including wanting to satisfy rising demand and reduce cost. Some participants, notably chefs, were motivated to provide more vegetable-forward dishes due to concerns over environmental sustainability due to animal product consumption. Study participants indicated that the demand for healthier offerings came largely from white (non-Hispanic) clients, but some recognized a growing market among young Hispanics. Cost-saving was discussed by chefs expressing interest in increasing vegetarian alternatives, noting the creative potential and the lower cost of vegetables compared to meats, along with the greater profit, as these were often sold at comparative prices. Additionally, one waitstaff mentioned the potential for up-selling healthier additions, such as side vegetables, as a potential to increase profit.

On the other hand, low acceptability for this strategy mainly came from concerns about demand, exacerbated by the uncertainty of COVID-19, pushing restaurants to focus on top sellers regardless of their relative healthfulness, or their own, personal interest in offering these items. The importance of perceived demand and notions about which offerings are perceived as “authentic” was also a reason that prevented some LARs from offering cuisines closer to contemporary healthier offerings in Latin America. For example, Mexico-born chefs discussed wanting to create dishes devoid of the cheese or sour cream typically found in Mexican-American (or “Tex-Mex”) cuisine but received pushback from the restaurant owners about the types of foods that should be offered in their restaurants. Some owners felt forced to include less healthy offerings due to perceived customer demand. Lastly, participants also discussed logistical issues regarding the storage and space requirements to accommodate healthier offerings. This included space for refrigeration and kitchen space and equipment capacity to diversify food preparation methods. Items such as salads or dishes containing seafood are perishable. They require high turnover or demand to avoid food waste – a concern that became more salient during the initial onset of COVID-19.

#### Providing nutrition information

Respondents were familiar with the provision of nutrition information in menus (Table 2) as a strategy associated with large corporate restaurants, especially fast-food restaurants. Only two respondents, coming from the same multi-location restaurant, had experience with this strategy, as one required by law. Acceptance of this strategy was associated with recognizing an increased interest in nutrition information. A few owners saw this as a possible way to make their restaurants stand out from the competition. Some were curious as to the nutrition content of the foods they served, expressing an openness for the analysis. However, some participants expressing high acceptability discussed the information as something to offer upon request instead of having it on full display. Participants also expressed not wanting to “rub that [nutrition information] in people’s faces” to avoid customers “feeling guilty about what they are eating” (waitstaff, Puerto Rican restaurant). While recognizing the benefit of the information was the main motivator driving some level of acceptance for this strategy, there was some pushback, particularly when the strategy did not fit with the restaurant concept or when additional resources were needed to provide the information. In these restaurants, recipes change frequently, and offerings were highly customized, factors that could make nutrient calculations inaccurate or require ongoing revisions, adding to the cost. Lastly, participants also expressed skepticism of whether the nutrition information made any difference in customer choice or, as expressed by one owner, whether such information might even trigger eating disorders.

#### Changing portion size

Portion size changes (Table 2) were discussed in two main ways – either a general decrease in the size of offerings or the offering of specific portion options, such as half-portions – the latter being the least accepted. Acceptability was found among those with experience with the strategy, seeing the benefit of offering half or smaller portions for business and customers. One respondent shared that the Mexican establishment he worked at experimented with smaller portions as part of their adjustments in response to COVID-19. The shift to a takeout mode of service created a motivation to create menu items that consisted of “small bite-sized things that [one could] just grab and go as quickly as possible.” In making this change, he noted, “We sell way more of the smaller stuff than we do the bigger orders.”

Low acceptability was primarily due to economic concerns. Some respondents argued that portions sizes resulted from estimations based on ingredient costs, potential profit, and perceived client expectations for larger portions. Thus, offering half-portions was seen as potentially detrimental for profit. Participants discussed expectations of check amounts per diner, particularly in full-service establishments, which might decrease if diners opted for half portions. The general sense was that the customer could always take the left-over home if they wished to do so. Additionally, the core ingredients in these dishes (e.g., white rice, beans, tortillas) were seen as largely inexpensive, and, hence, not really motivating for restaurateurs to decrease portion sizes to save money. Some also expressed concerns over challenging logistics and additional costs related to this HEPS, particularly in the pricing calculation for the half-portion and the need for menu re-design.

### Resource needs for engaging in HEPS

The discussions revealed a high level of operational burden affecting LAR owners that could prevent them from making innovations in support of healthier eating. They shared several resource needs, notably help with restaurant promotion and assistance in accessing information about local regulations and complying with them (should certain HEPS become law). Table 3 summarizes the perceived resources needed in relation to the HEPS discussed. Nutrition knowledge as a resource was seen as common across all HEPS, except for portion size change. The knowledge can help in the identification of offerings to highlight or promote and in the development of new, healthier offerings. Design expertise was identified as a need to facilitate menu design and promotion activities to convey new offerings in a clear and appealing fashion.

**Table 3:** Identified resource needs by HEPS, n (%), *N* = 20

Identified resource needed:	Menu highlights	Promotion of healthier choices	Increase healthier choices	Provision of nutrition information	Reduced portion size
Nutrition knowledge	1 (5%)	2 (10%)	2 (10%)	4 (20%)	
Design expertise	2 (10%)				2 (10%)
Social media expertise		2 (10%)			
Well-trained staff		2 (10%)			
Latin cuisine promotion		7 (35%)			
Consumer nutrition education		7 (35%)			
Culinary expertise/assistance with recipe development			5 (25%)		
Latin cuisine research			5 (25%)		
Food supplier research and connections			5 (25%)		
Policy incentives			3 (15%)		
New packaging/serve ware					1 (5%)

The strategies with the most identified resource needs were the increase of healthier offerings and the promotion of these items. Respondents noted the need for social media expertise, including an expert to craft messaging and create appealing imagery for highly visual and popular platforms, like Instagram. They also noted the need for general promotion concerning Latin cuisines. Respondents discussed a lack of general knowledge among clients, including notions that LARs were generally unhealthy or inaccurate views concerning the healthfulness of specific offerings, for example, negative views about vegetarian options, including the perception that “meat is really good for you, and if you don’t eat meat, you’re going to get sick.” (Waitstaff, full-service Mexican restaurant, NYC). Related to this, some mentioned the need for community nutrition education, in general, and at schools to make children aware of healthier eating, as well as greater dissemination or promotion of healthier eating trends, such as vegetarian and pescatarian diets.

When asked about resource needs for the provision of healthier menu offerings, these included additional culinary training or tips to develop new, palatable, healthy choices, including assistance in recipe development. In our sample, chefs who already actively engaged in cooking healthier dishes took the initiative to research the ingredients and cuisines, including travels to Latin America. However, this is not accessible to most chefs or cooks, necessitating alternative ways to access the information. As described by one of these chefs, this could include a list of traditional produce and condiments for other chefs to experiment with. At the same time, others mentioned the need for creativity and an open mind about the cuisines – which were key characteristics among the chefs already engaged in developing innovative, healthier dishes in LARs. In addition, some participants noted the need to increase awareness among owners and chefs about the need to provide healthier foods and how this change could lead to better business outcomes. Another important resource needed was assistance with food sourcing. For example, one owner mentioned wanting assistance with food supplier research to facilitate the identification of local food suppliers that offered competitive pricing for healthier ingredients. Some also mentioned interest in developing connections with local food producers, which could result in fresher produce and new dishes based on producers’ perspectives. Lastly, participants also mentioned policy factors. While the participants shared the perception that the sector was over-regulated, they discussed the need to provide policy-level incentives to increase healthier offerings. These included monetary incentives for restaurants to incorporate local produce or producers to work with restaurants. One chef mentioned the idea of creating an incentive tied to certifications or permits or providing a tax incentive for restaurants that offer training on local produce to their staff.

While the least accepted, reducing portion size was associated with fewer resource needs. Counter-style restaurants would likely require new packaging for smaller portions. A second less pressing resource was the need for design expertise to assist with menu re-design to accommodate the half-portion offering in a visually appealing and understandable format.

## Discussion

Restaurants have an increasingly vital role in the facilitation of healthy eating practices. However, research has been limited regarding business perspectives on improving the consumer nutrition environments in these establishments. This research presented key perspectives from the LAR sector and found different levels of acceptability for potential HEPS. The HEPS that were viewed more favorably – menu highlights, promotion of healthy choices, and increasing healthy options – have shown some evidence of effectiveness in community-based (non-corporate) restaurants, especially when combined in interventions [12]. Acceptability was higher for strategies that facilitated or promoted foods, such as menu highlights and promotion efforts, with acceptability linked to perceived beneficial business outcomes. Food promotion was generally seen as good for business, which has been the focus of most interventions, particularly in non-corporate restaurants [5, 12]. While these past efforts have emphasized on-site promotion (i.e., tents placed at restaurant entrance or signs at point-of-purchase), our research revealed the growing importance of virtual spaces (social media) for promotion efforts, a focus that was seen as more critical with the onset of COVID-19 [23].

Increasing the options of healthier offerings was the third most accepted strategy, despite being one that required the most resources, including investments in ingredients and recipe development. The provision of healthier foods was overall discussed positively, and this HEPS has been successfully implemented in various restaurants [5]. Our discussions revealed a generally high level of acceptability, but this was discussed largely as offering more choices for vegetarian consumers or the potential to provide more seafood options. Markedly, additions were discussed at greater length as opposed to decreasing potentially unhealthy options, such as fried foods, signaling that these menu items would stay on menus as long as restaurants perceived demand for these items; this finding is consistent with other research [24]. For restaurants serving immigrant or “ethnic” cuisines, the perceived demand is also influenced by notions of authenticity, or the highly subjective views of which dishes or ways of cooking are deemed essential.

The two least accepted HEPS, the provision of nutrition information and offering smaller portion sizes, are noteworthy. The provision of nutrition information has been one of the most documented HEPS in research and a focus for regulation among corporate restaurants. The resistance to this HEPS echoes what has been documented in past research with independently owned restaurants, notably issues with feasibility and costs associated with providing accurate nutrition information [25]. One respondent expressed resistance to this strategy, due to its potential to exacerbate eating disorders. This concern has been addressed by researchers showing that nutrition labels may negatively impact calories consumption among those diagnosed [26]. This concern should be considered alongside research that shows that menu labeling yields mixed or weak effectiveness in changing customer ordering behavior [27–29]. At the same time, this HEPS also has the potential to motivate recipe changes to reduce calorie, fat, and sodium content in dishes [28]. The resistance documented in this research shows the need to collaborate with the restaurant sector to develop innovations to make menu labeling more accessible and feasible among LARs and other independently-owned restaurants. For example, it would be useful to develop easy-to-use technology for restaurants to do their own nutrition analysis while having the ability to integrate such a function with existing software for monitoring food and recipe costs, recipe management, inventory, budget, sales, and forecasting. These can come in the form of culinary software services or using the point of sales systems to analyze menu items and sales volumes. The use of software, while not accessible to all restaurants, is increasingly common, especially as restaurants have been adapting to delivery through online third-party services.

Contrary to menu labeling, portion control or reduction has not been the focus of existing regulation, with some notable exceptions, such as the failed sugary beverage size cap in NYC [30]. Research has shown the association between portion sizes and obesity, as well as the lack of reduction in portions in light of this research [31]. Our study shows the importance of perceived social norms regarding portion size expectations as a key barrier for this HEPS and the importance of revenue expectations based on estimated plate costs per customer. While the ingredient cost may be lowered if reduced portions are offered, the savings are not perceived as enough compared with the potential revenue loss per customer. These barriers are complex, illustrating the need for more research on strategies that include food presentation (e.g., serving dish shape and color) [32] or increases in the relative portion size of healthier items such as vegetables vs. high energy items such as fatty meats [33]. Additionally, the provision of portion choices can be explored further through peer modeling interventions, where owners can learn how to implement this HEPS from their peers. The respondents who had experience with portion size management showed a high level of acceptance of reduced portion sizes because they recognized the benefit to consumers without adverse effects on the businesses’ bottom line.

### Research strengths and limitations

This study was strengthened by our user-focused approach, prioritizing the needs and perspectives from the restaurants and engaging members of the sector as part of the research team. This approach allowed us to adapt the study to incorporate COVID-19 as an essential context for our discussion. COVID-19 led to many changes in the industry that both promoted and hindered the implementation of HEPS. The pandemic forced changes and adjustments and, for some, increased the importance of health. However, it also added to the already high operational burden, where owners have to contend with changing regulations, balancing staffing needs, and catering to customer demand. Hence, COVID-19 serves as both motivation and barrier for the engagement in HEPS and participation in this research and had an inevitable influence in the responses gathered. Our use of virtual discussions allowed for a greater reach to participants and decreased the cost of participation (e.g., transportation time for in-person meetings). While COVID-19 forced many restaurants into virtual spaces, our approach inevitably excluded those without access to the internet, computers, or technical know-how. We sought to facilitate this by providing information about our main platform (Zoom) as part of the scheduling communication, including the alternative to join by telephone, if preferred. Still, we had 26% (*n* = 7) of recruited participants as “no shows” to interviews. We followed up to reschedule interviews, but with no response. While the reason for these no shows was not discerned, our informal conversations with participants revealed that the changing and demanding nature of restaurant work might have been the primary cause for attrition. Long work hours and schedule changes at the last minute might have decreased motivation for participation. Hence, our results are limited to the perceptions of those that had the interest and capabilities to participate in this study. While the study provides valuable information on HEPS acceptability, these results must consider that acceptability, in theory, may not readily translate to action, given the operational constraints restaurants face.

## Conclusions

The improvement of consumer nutrition environments in LARs requires acknowledging and meeting owners’ needs to both sustain and increase revenue while making changes to promote healthier eating. The examination of the business perspective provides much-needed nuance to public health literature addressing restaurant-based interventions. While most of the research has focused on customer behaviors, this research examined the perspectives from LARs, including three distinct stakeholders: Owners, chefs/cooks (“back of the house”), and waitstaff (“front of the house”). LARs represent a sector in the industry with the potential to change social and cultural norms to address persistent diet-related health inequities among Latin communities and beyond. LARs face additional challenges, given the immigrant composition of the owners and workforce that prevent them from accessing assistance and services. At the same time, they are bound by social norms, influenced by cuisine authenticity, which prevents some from wanting to engage in HEPS.

Our research contributes to a growing body of work that engages restaurants as critical institutions in community food environments. Future research is needed to expand on this work. Future research avenues include examining the influence of other sectors and stakeholders, including food suppliers, as well as factors that influence social norms concerning expectations for LARs. The latter should incorporate the impact of food media, opinion leaders, and interactions with other restaurants in motivating or hindering the implementation of new HEPS. While policy was identified as a key aspect to motivate HEPS implementation, future research can find ways to use policy as a carrot rather than a stick, leading to the exploration of incentive-based changes, as opposed to regulation or penalty-focused policies to best serve small, community-based businesses and help them contribute to improving community health.

This study aimed to enhance our understanding of LARs and their ability to promote healthier eating. A better understanding of the sector can facilitate increased collaboration and evidence generation for future policies and interventions. LARs have the potential to facilitate healthier eating within the communities they serve, but engagement with the sector requires balancing public health goals and business profitability. This balancing act requires acknowledging and addressing the multiple competing priorities LARs face, requiring different levels of assistance and resources to promote these changes, underscoring the need for innovative engagement approaches and incentives to promote these changes.

## Data Availability

The qualitative data used in this study is not publicly available to protect the confidentiality of respondents. De-identified data and materials supporting findings are available upon reasonable request from the corresponding author.

## Abbreviations

COVID-19: Coronavirus Disease 2019
HEPS: Healthy eating promotion strategies
LAR: Latin American Restaurants
NYC: New York City
US: United States
